# Spatial heterogeneity of knockdown resistance mutations in the dengue vector *Aedesalbopictus* in Guangzhou, China

**DOI:** 10.1186/s13071-022-05241-7

**Published:** 2022-05-03

**Authors:** Xueli Zheng, Zihao Zheng, Shanshan Wu, Yong Wei, Lei Luo, Daibin Zhong, Guofa Zhou

**Affiliations:** 1 Department of Pathogen Biology, School of Public Health, Southern Medical University, Guangzhou, Guangdong China; 2 Guangzhou Center for Disease Control and Prevention, Guangzhou, China; 3 Program in Public Health, School of Medicine, University of California, Irvine, CA USA

**Keywords:** *Aedesalbopictus*, Insecticide resistance, Voltage-gated sodium channel gene, Haplotype frequency, Guangzhou

## Abstract

**Background:**

The city of Guangzhou has been the epicenter of dengue fever in China since the 1990s, with *Aedes* *albopictus* being the primary vector. The main method used to control vectors and prevent dengue fever has been the application of chemical insecticides; however, this control strategy has resulted in the development of resistance to these insecticides in mosquitoes. Here we report our investigation of the patterns of knockdown resistance (*kdr*) mutations in 15 field populations of *Ae.* *albopictus* collected from 11 districts in Guangzhou.

**Results:**

Four mutant alleles (V1016G, F1534S, F1534C, F1534L) were detected in domain II and III of the voltage-gated sodium channel (VGSC) gene. Various allele frequencies of *kdr* mutations were observed (3.1–25.9% for V1016G, 22.6–85.5% for F1534S, 0–29.0% for F1534L, 0.6–54.2% for F1534C). Seven *kdr* haplotypes (VF, VS, VL, VC, GF, GC, GS) were identified; the highest frequency of haplotypes was found for the single mutant haplotype VS (50.8%), followed by the wild-type VF haplotype (21.7%) and the single mutant haplotype VC (11.9%). Of the three double mutant haplotypes, GF was the most frequent (8.8%), followed by GC (1.2%) and GS (0.8%). *Aedes* *albopictus* showed spatial heterogeneity in deltamethrin resistance in populations collected in Guangzhou. We also observed significant differences in haplotype frequency. The frequency of the VC haplotype was significantly higher in high-risk dengue areas than in low-risk ones.

**Conclusions:**

The *kdr* allele V1016G was discovered for the first time in Guangzhou. Genetic isolation in mosquito populations and long-term insecticide selection seem to be responsible for the persistent, patchy distribution of *kdr* mutant alleles. The small-scale spatial heterogeneity in the distribution and frequency of *kdr* mutations may have important implications for vector control operations and insecticide resistance management strategies.

**Graphical Abstract:**

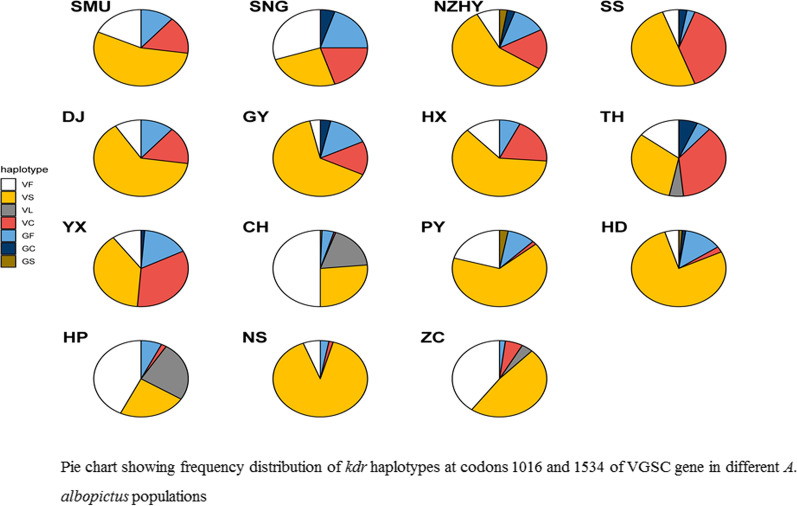

**Supplementary Information:**

The online version contains supplementary material available at 10.1186/s13071-022-05241-7.

## Background

The Asian tiger mosquito *Aedes* *albopictus* (Skuse) is a highly invasive mosquito species that is distributed in > 70 countries worldwide. It is a semi-domestic mosquito species and shows a high adaptability to diverse climate and ecological environments. It is the main vector of five arboviruses: dengue virus, chikungunya virus, Zika virus, Rift Valley fever virus and yellow fever virus [1]. Although dengue fever transmitted by *Aedes* *aegypti* represents a greater challenge to public health, leading to larger, more serious epidemics, than dengue fever transmitted by *Ae.* *albopictus* [2], the impact of *Ae.* *albopictus* on human society cannot be neglected. *Aedes* *albopictus* is the most invasive and widely distributed mosquito species in China, ranging from Hainan Province in the south to Dalian Province in the north. In comparison, *Ae.* *aegypti* is only found in Hainan and Yunnan provinces and in a small area of the southernmost part of Guangdong Province [3]. In recent years, it has been reported that China was originally dominated by *Ae.* *aegypti*, but *Ae.* *albopictus* has now become the leading species and possibly the main vector of dengue fever in mainland China [4].

Vector control, one aspect of which is reducing the density of vector insects, is an important strategy for preventing and controlling vector-borne epidemics [5–7]. The development of mosquito larvae can be controlled by preventing the accumulation of standing water in containers such as flowerpots, basins or cylinders, or by using mosquito larvicides to eliminate mosquito breeding habitats. However, these methods are inadequate for controlling adult mosquitoes during a disease outbreak when it is pivotal to use pyrethroid insecticides or organic phosphate insecticides for space spraying [8]. The continuous and intensive use of such insecticides in fields or domestic settings can, however, artificially create a direct or indirect selection pressure on vector insects, eventually leading to the development of insecticide resistance (IR) [1, 9]. Insecticide resistance has become a major impediment to achieving control of mosquito-borne diseases worldwide. To tackle this issue, the WHO launched the Global Plan for Insecticide Resistance Management to guide rational insecticide usage [10, 11]. Understanding the mechanisms underlying IR in vector populations is crucial for effective IR management.

The two foremost mechanisms of IR are alterations in target sites and increases in insecticide metabolism rate [12, 13]. While metabolic resistance is primarily caused by cytochrome P450s, esterases and glutathione S-transferases, target site resistance is conferred by one or many mutations in insecticide target sites [12, 13]. Target site resistance occurs mainly due to nonsynonymous mutations in one or more genes encoding voltage-gated sodium channels (VGSCs), resulting in changes in their configuration. Mosquitoes show varying degrees of resistance owing to their different sensitivities to the various pyrethroid insecticides [9, 14]. This phenomenon is commonly known as knockdown resistance (kdr) [15].

Lan et al. analyzed VGSC gene mutations in *Ae.* *albopictus* populations showing resistance to pyrethroid insecticides in Ruili City, Yunnan Province (China) and found mutations at codons 1532, 1534 and 1763, but mainly at codon 1534 [16]. Zhu et al*.* studied the distribution of *kdr* genotypes in *Ae.* *albopictus* in Jinghong City, Yunnan Province from 2018 to 2019 and reported the presence of mutations in the V1016, I1532 and F1534 genes [17]. Further, Zhou et al. reported the presence of multiple mutations (V1016G, I1532T, F1534L/S) in VGSCs in *Ae.* *albopictus* populations in Beijing [7]. Liu et al. reported the I1532T, F1534L and F1534S mutations in domain III of the VGSC gene of *Ae.* *albopictus* in Shangdong [18]. F1534S and F1534L mutations have also been reported in VGSCs. Li et al. observed the existence of mutations in IR mosquitoes and in urban *Ae.* *albopictus* populations in southern China, reporting that the F1534S and F1534L mutations were significantly associated with deltamethrin resistance [19].

Guangzhou, the largest city in southern China and a district of Guangdong Province, has been the epicenter of dengue fever since the 1990s. *Ae.* *albopictus* has been reported to be the only vector of dengue fever in Guangzhou [20]. There have been many outbreaks of dengue fever in Guangdong, Hainan and Zhejiang provinces. In 2014, 45,203 cases of dengue and six deaths due to dengue were reported in Guangdong Province, of which 99.8% were local cases; these cases accounted for 96% of the total cases of dengue in China [21, 22]. No specific antiviral treatment is available as yet, and vaccines against dengue need to become more effective. Thus, vector control continues to remain the best method to prevent and control dengue fever [23]. In 2014, with the aim to tackle the dengue epidemic in Guangzhou, pyrethroid insecticides were applied on a large scale to kill adult mosquitoes; this strategy also included the intensive use of the organophosphates parathion and fenthion to kill mosquito larvae. At that time, insecticides were sprayed on > 3291 km^2^ of land, accounting for 44.3% of the total area of Guangzhou [24, 25].

During this period, insecticides were sprayed more intensely and more frequently in urban areas than in suburban and rural areas, and it has since been observed that adult *Ae.* *albopictus* populations in urban areas are more resistant to pyrethroids than those in rural areas [26]. From 2015 to 2017, Su et al. collected *Ae.* *albopictus* populations and found different dengue virus transmission intensities from four districts in Guangzhou. These authors used the standard WHO tube tests to determine insecticide susceptibility to deltamethrin (0.03%), permethrin (0.25%), dichlorodiphenyl trichloroethane (4%), malathion (0.8%) and bendiocarb (0.1%) in adult *Ae.* *albopictus*, and found that *Ae.* *Albopictus* populations [11, 26, 27] rapidly developed a very high resistance to multiple commonly used insecticides in all study areas [25, 28–31]. Further, Li et al. reported that adult *Ae.* *albopictus* populations in Yuexiu District had developed resistance to dichlorodiphenyl trichloroethane and deltamethrin [26]. Such reports of IR in Guangzhou raise serious concerns about the efficacy of chemical insecticides against *Ae.* *albopictus* and the effectiveness of the curent dengue transmission control policy in China.

We report here our investigation of the distribution pattern of kdr mutations in 15 field populations of *Ae.* *albopictus* collected from 11 districts in the city of Guangzhou, with the aim of gaining insights into resistance management and identifying suitable methods to prevent and control this important dengue vector.

## Methods

### Study sites and sampling

**Fig. 1:**
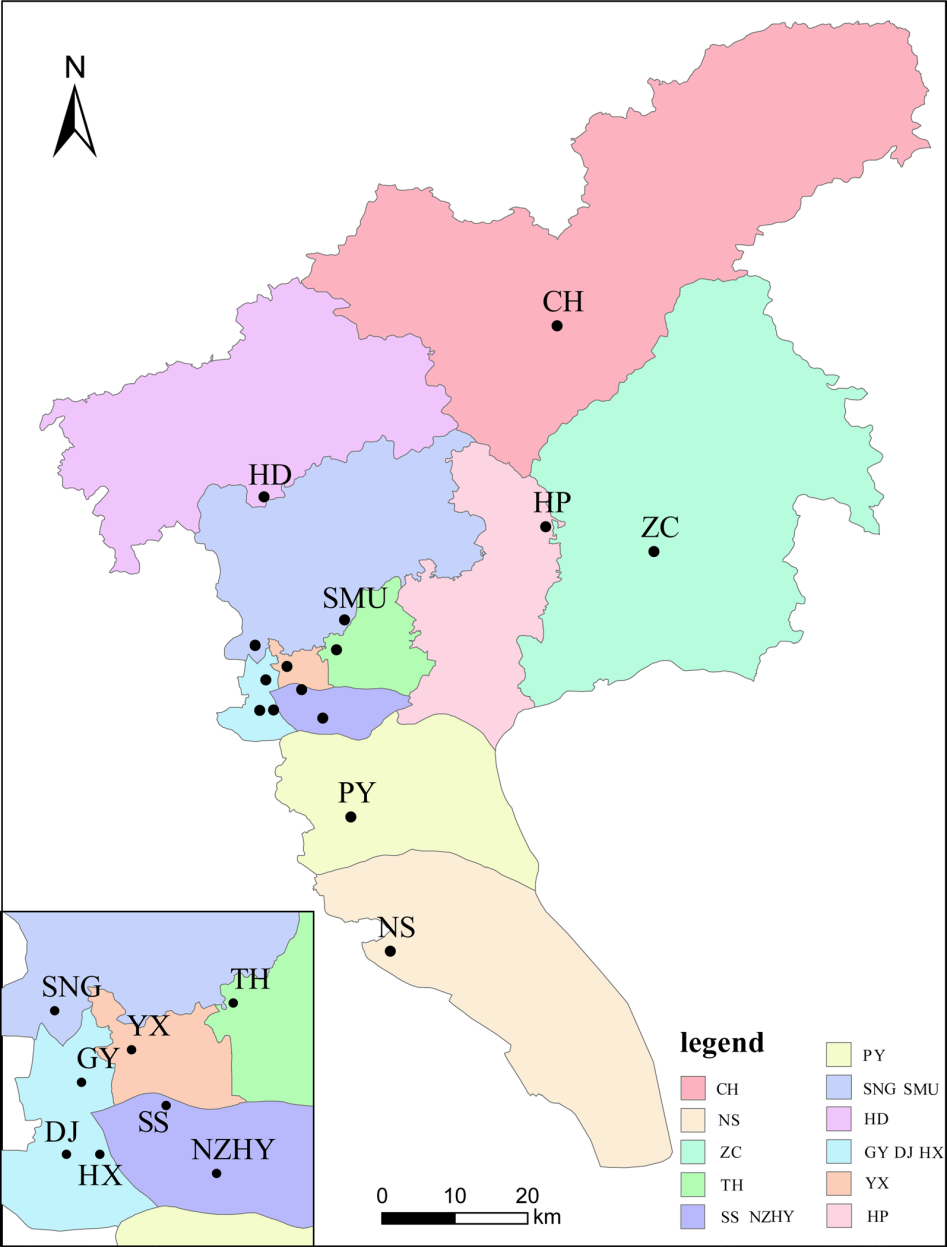
Map showing the sampling sites of *Ae.albopictus* in Guangzhou, China. Abbreviation: VF, VS, VL, VC, GF, GC, GS, 7 knockdown resistance (*kdr*) haplotypes identified in this study; V/V, G/G, V/G, wild-type homozygote, mutant homozygous and wild/mutant heterozygote, respectively. *CH* conghua district, *HD* huadu district, *HP* Huangpu distict, *ZC* zengcheng district, *SMU* southern medical university, *PY* panyu district, *NS* nansha district, *SNG* songnange (baiyun district), *TH* tianhe district, *YX* yuexiu district, *GY* the third affiliated hospital of Guangzhou Medical University, *DJ* dongtunsanqiaofang (Liwan District), *SS* sushe (Haizhu District), *HX* hexiang (Liwan District), *NZHY* nanzhouhuawan (Haizhu District)

From July 2020 to November 2020, *Ae.* *albopictus* populations were collected in 15 sites of 11 administrative regions (districts) in the city of Guangzhou (Fig. 1). The study sites in the districts of Baiyun, Haizhu, Tianhe, Liwan and Yuexiu are places with a large population flow and developed economies; in comparison, those in the districts of Conghua (CH), Panyu (PY), Huadu (HD), Huangpu (HP), Nansha (NS) and Zengcheng (ZC) are places with a relatively small population flow and with many surrounding villages. The average distance based on GPS locations between the sites was 25.5 ± 17.3 km (range: 1.5–76.5 km). Detailed information on the sampling sites is given in Additional file 1: Table S1. Larvae were collected using Pasteur pipettes from different habitats, including disposable plastic containers, tires and water containers. No more than five larvae were collected per habitat to reduce potential bias from collecting mosquito full siblings. The larvae were then reared to adults under laboratory conditions (28 °C ± 1 °C; 70% ± 10% relative humidity; 14/10-h day/night photoperiod). Adult mosquitoes were captured via the human-baited double net trap and human landing catch [32].

### Species identification

All adult mosquitoes were morphologically identified using the method reported by Lu et al. [33]. In brief, the mosquitoes were soaked in absolute alcohol and stored at − 80 °C until analysis. Genomic DNA was extracted from a single mosquito using the Insect DNA Kit (Omega Bio-Tek, Norcross, GA, USA), according to the manufacturer’s instructions and stored at − 80 °C for subsequent use. PCR tests was performed using the extracted genomic DNA to amplify mitochondrial cytochrome* C* oxidase (COI) genes; the primers for amplifying COI gene fragments refer to the general primers used by Folmer et al. [34, 35] (forward primer: 5′-ggtcaaaataagatattgg-3′; reverse primer: 5′-tgatttttggtcacccctgaagtta-3′). Sequencing was performed by Qingke Biotechnology Co., Ltd. (Beijing, China) using a reverse primer. The Basic Local Alignment Search Tool (BLAST) was used to compare the sequences against those in GenBank to confirm species identity [35]. Analysis of the genetic diversity of 15 *Ae.* *albopictus* populations in Guangzhou based on the mitochondrial COI gene has been published [35].

### Amplification of partial VGSC genes

Point mutations in the VGSC gene were analyzed in a 25-µl reaction mixture containing 12.5 µl premix Taq (TaKaRa, Beijing, China), 1 µl forward primer, 1 µl reverse primer (Additional file 2: Table S2), 2 µl genomic DNA template (extracted as described above) and sterilized ddH2O. The cycling conditions were: 94 °C for 2 min, followed by 35 cycles of 94 °C for 30 s, 55 °C for 30 s and 72 °C for 30 s, with a final extension at 72 °C for 8 min. The amplicons were purified by gel electrophoresis and sequenced using the primers listed in Additional file 3: Table S3 by Qingke Biotechnology Co., Ltd. Primers for sequencing were designed with reference to those used by Kasai et al. [36].

### Data analysis

The sequences were first converted from AB1 files to FASTA files using the EditSeq 7.1.0 program in the Lasergene software package (DNASTAR, Madison, WI, USA). Genotypes were read by BioEdit 7.2.5 [37] and then documented by Excel (Microsoft Corp., Redmond, WA, USA). *kdr* haplotype analysis was achieved using PHASE (https://stephenslab.uchicago.edu/phase/download.html), and multivariate clustering analysis was performed using PAST (https://www.nhm.uio.no/english/research/infrastructure/past/). Pie charts of genotypes and alleles were drawn using R version 4.0.3 [38] using the “ggplot2” [39] and “cowplot” [40] packages. The SPSS 2020 software package (SPSS IBM Corp., Armonk, NY, USA) was used to analyze the allele and genotype frequencies of VGSC genes. Only sequences showing a monoclonal peak in both the exon and intron regions were used for phylogenetic tree construction. Initially, the sequences including the exon and intron regions were aligned in MEGA-X [41] with the MUSCLE [42] algorithm. Haplotype identification was achieved using DNAsp version 6.12.03 [43]. The best model for phylogenetic tree construction was determined using the “Find Best DNA/Protein Models (ML)” option based on the lowest Bayesian information criterion in MEGA-X. The sequences of one* Ae.aegypti* strain and several* Ae.albopictus* strains from different geographical locations from the gene library were received as follows: One* Ae. aegypti* sequence (accession no.: domain II KM677332.1, Beijing MK201608.1, Japan AB828338.1 and AB827810.1, Brazil KX281169.1 and KX281170.1 and Nepal LC485547.1; domain II Beijing MK201621.1 MK201630.1, and MK201631.1; HaiKou MH384961.1; and ShangHai MH384958.1).

## Results

### Type of *kdr* mutations in *Ae. albopictus* VGSC gene

**Fig. 2:**
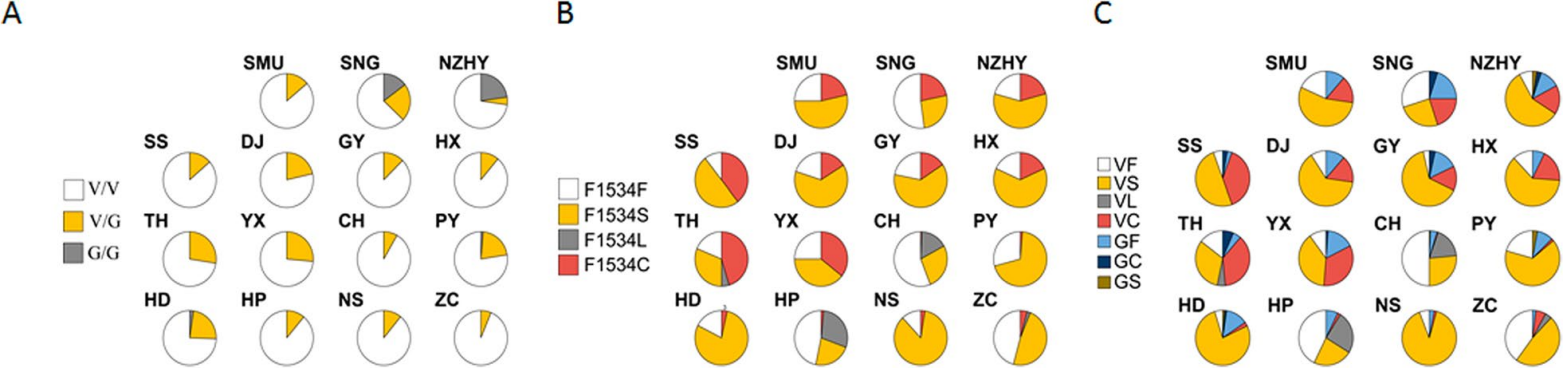
Nonsynonymous mutations at loci 1534 and 1016 of the voltage-gated sodium channel (VGSC) gene in *Ae.* *albopictus.* **a** Codon 1534, **b** codon 1016

Overall, 659 sequences from domain II, 654 from domain III and 706 from domain IV were obtained for analysis in this study. The *kdr* loci in *Ae.* *albopictus* were coded according to the VGSC gene of *Musca* *domestica.* Nonsynonymous mutations were found at codon 1016 of domain II and at codon 1534 of domain III in all the 15 field populations of *Ae.* *albopictus* collected in Guangzhou (Fig. 2a, b). No nonsynonymous mutations in the VGSC gene were found in codons 1011 and 1014 of domain II, codon 1532 of domain III and codon 1763 of domain IV. Two alleles were found at codon 1016 of domain II, namely the wild-type allele V1016V and the mutant allele V1016G. GTA was the core base sequence of the V1016V allele. Only one wild-type heterozygous GTA/GTG (1/659) was found among the mosquitoes collected in Huangpu District; three genotypes were identified: wild-type homozygous V/V (GTA/GTA and wild-type heterozygous GTA/GTG); wild/mutant heterozygous V/G (GTA/GGA); and mutant homozygous G/G (GGA/GGA). Four alleles were found at codon 1534 of domain III, namely F/F, F/S, F/C and F/L, corresponding to the wild-type allele F1534F and mutant alleles F1534S, F1534L and F1534C, respectively. TTG was the core base sequence of allele F1534L, and only two cases of CTC were found in Conghua district; the genotype was wild homozygous F/F (TTC/TTC). Wild/mutant heterozygotes F/S (TTC/TCC), F/C (TTC/TGC), F/L (TTC/TTG, TTC/CTC); Mutant homozygous L/L (TTG/TTG), S/S (TCC/TCC), C/C (TGC/TGC); Mutant heterozygote S/C (TCC/TGC) (Additional file 4: Fig. S1).

### Frequency and distribution of *kdr* alleles in *Ae. albopictus* VGSC gene

The mutant allele V1016G was found in all 15 wild-type populations of *Ae.* *albopictus *collected in Guangzhou, with a frequency ranging from 3.1% to 25.9% (Table 1; Fig. 2). The majority of the genotypes were wild-type V/V (82.8%), followed by the wild-type/mutant V/G (16.0%); the mutant allele G/G was presented at a frequency of only 1.2%. The mutant homozygous allele G/G was only found in the SNG (14.8%), NZHY (4.5%), PY (1.4%) and HD (2.3%) populations. The mutant allele F1534S was found in all the 15 populations, raning from 22.6% (HP) to 85.5% (NS); furthermore, the mutant allele F1534L was found only in four populations: TH (4.7%), CH (16.5%), HP (29.0%) and ZC (2.1%). Further, the mutant allele F1534C was found in all the 15 populations, ranging in frequency from 0.6% (CH) to 45.3% (TH). In general, F1534S (frequency: 45.2%) was the predominant mutant allele, followed by F1534C (11.2%); F1534L was the least prevalent (3.9%, 51/1308). The frequency of mutated genotypes ranged from 44.1% (CH) to 89.5% (SS) (Table 2; Fig. 2).

**Table 1:** Frequency and distribution of genotypes and alleles at codon 1016 of the voltage-gated sodium channel (VGSC) gene in the field populations of *Ae. albopictus*

Collection site^a^	*n*	Frequency				
		Genotype (%)			Allele (%)	
		V/V	G/G	V/G	V	G
SMU	36	86.1	0	13.9	93.1	6.9
SNG	27	63	14.8	22.2	74.1	25.9
NZHY	44	72.7	4.5	22.7	84.1	15.9
SS	22	86.4	0	13.6	93.2	6.8
DJ	28	78.6	0	21.4	89.3	10.7
GY	16	87.5	0	12.5	93.8	6.3
HX	27	88.9	0	11.1	94.4	5.6
TH	40	72.5	0	27.5	86.3	13.8
YX	53	73.6	0	26.4	86.8	13.2
CH	109	91.7	0	8.3	95.9	4.1
PY	71	77.5	1.4	21.1	88	12
HD	43	74.4	2.3	23.3	86	14
HP	36	89.1	0	10.9	95.8	4.2
NS	55	89.1	0	10.9	94.5	5.5
ZC	32	93.8	0	6.3	96.9	3.1

V, Wild-type allele; G, mutant allele; V/V, wild-type homozygote; G/G, mutant homozygous; V/G, wild/mutant heterozygote

^a^SMU, Southern Medical University; SNG, Songnan Pavilion; NZHY, Nanzhou Garden; SS, Su she; DJ, Dongyi sanqiaofang; GY, The Third Affiliated Hospital of Guangzhou Medical University; TH, Tianhe Villa; YX, Yue Xiu community; CH, No. 7 middle school in Conghua district; PY, Pan Yu Xiaoping Village; HD, Hua Du Dongjing Village; HP, Huang Pu Xintian Village; NS, Nan Sha Tanzhou Park; ZC, Zeng Cheng Nangang Village

**Table 2:** Frequency and distribution of genotypes and alleles at codon 1534 of the VGSC gene in the 15 *Ae.* *albopictus* field populations

Collection site	*n*	Frequency (%)											
		Genotype								Allele			
		F/F	F/S	F/L	F/C	S/S	L/L	C/C	S/C	F	S	L	C
SMU	28	7.1	25		10.7	35.7		10.7	10.7	25	53.6		21.4
SNG	23	47.8			8.7	13		4.3	26.1	52.2	26.1		21.7
NZHY	41	4.9	19.5		12.2	39		4.9	19.5	20.7	58.5		20.7
SS	19	5.3	5.3		5.3	36.8		26.3	21.1	10.5	50		39.5
DJ	25		32		8	40		4	16	20	64		16
GY	16		43.8			25			31.3	21.9	62.5		15.6
HX	22	13.6	9.1			50		9.1	18.2	18.2	63.6		18.2
TH	32		9.4	3.1	25	12.5	3.1	18.8	28.1	18.8	31.3	4.7	45.3
YX	42	7.1	16.7		19	19		14.3	23.8	25	39.3		35.7
CH	85	28.2	24.7	30.6		14.1	1.2		1.2	55.9	27.1	16.5	0.6
PY	79	19	20.3			58.2			2.5	29.1	69.6		1.3
HD	43		32.6		2.3	60.5			4.7	17.4	79.1		3.5
HP	31	22.6	22.6	25.8		9.7	16.1		3.2	46.8	22.6	29	1.6
NS	38		23.7			71.1			5.3	11.8	85.5		2.6
ZC	48	18.8	50	4.2		18.8			8.3	44.9	47.4	2.6	5.1

F, Wild-type allele; S, L and C, mutant alleles; F/F, wild-type homozygote; S/S, L/L and CC, mutant homozygous; F/S, F/L, F/C, wild/mutant heterozygote; S/C, mutant/mutant heterozygote

### The frequency of double-codon haplotypes

**Fig. 3:**
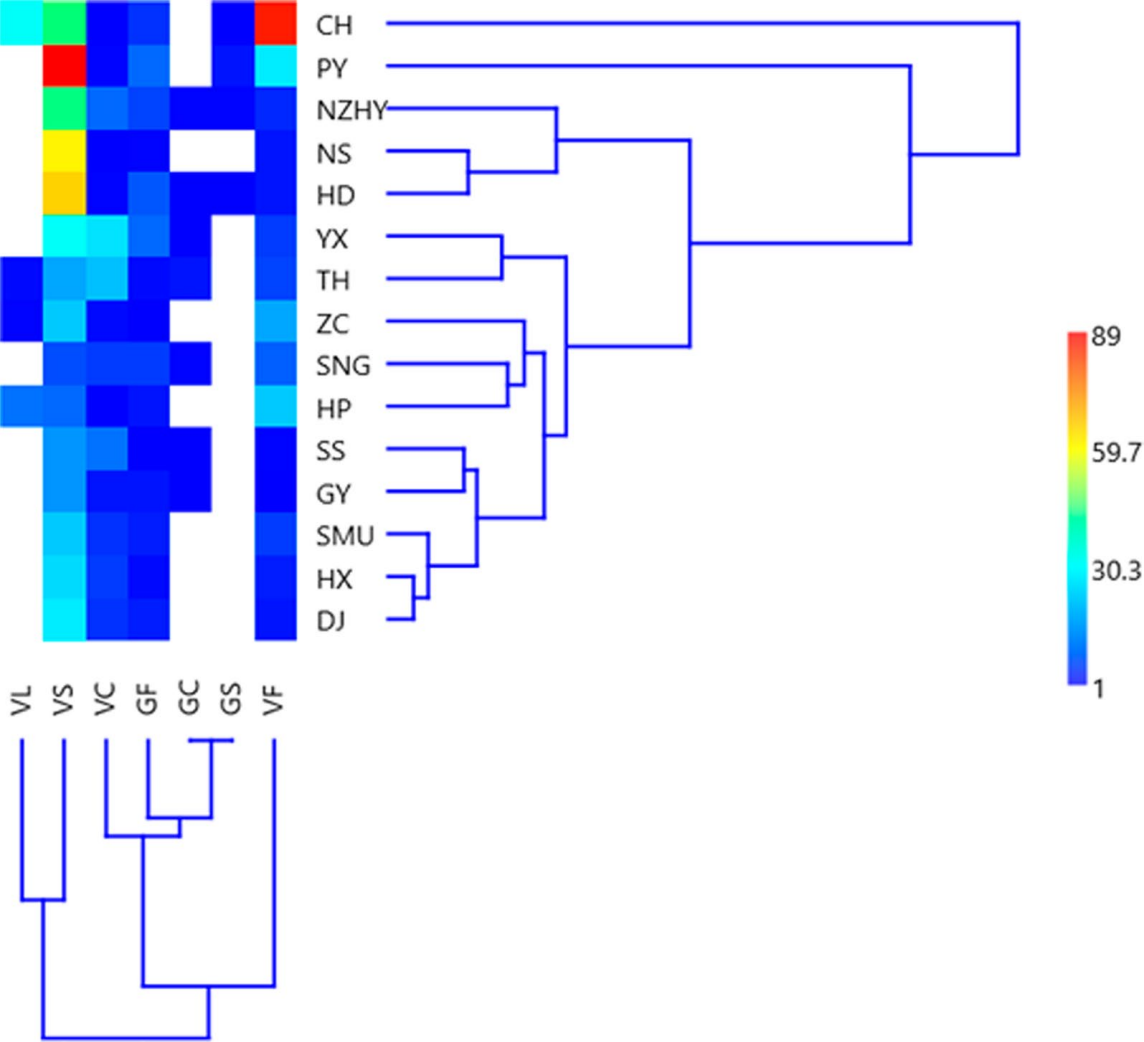
Pie chart showing frequency distribution of *kdr* haplotypes at codons 1016 and 1534 of the VGSC gene in different *Ae.* *albopictus* populations

Seven haplotypes (VF, VS, VL, VC, GF, GC, GS) were identified upon combining the two target codons at codons 1016 and 1534 of the VGSC gene (Fig. 2). The highest frequency of haplotypes was found in the single mutant haplotype VS (50.8%), followed by the wild-type VF (21.7%) and single mutant haplotype VC (11.9%). Three mutant haplotypes showed the highest frequency: GF (8.8%), GC (1.2%) and GS (0.8%). Significant differences in haplotype frequency were observed among populations (Pearson’s Chi-squared test: *χ*^2^ = 569.7, *df* = 84, *P* < 0.0001). Multivariate clustering analysis showed three patterns of haplotype frequency distribution among populations: cluster 1 (VF), cluster 2 (VC, GF, GC, and GS) and cluster 3 (VL and VS) (Fig. 3). The haplotype frequencies were very much different in the CH and PY populations than in the other populations, although the haplotype frequencies of the PY population appeared to be close to that of the other sites (Figs. 1, 3). The frequency of the VC haplotype was significantly higher in high-risk dengue areas than in low-risk ones (analysis of variance: *F*_1,13_ = 25.9, *P* < 0.0002).

### Evolutionary analysis of *kdr* mutations at codons 1016 and 1534

On PCR amplification, the domain II fragment produced a partial sequence of exon 20 (168 bp) of the *Ae.* *albopictus* VGSC gene, full-length sequence of intron 20 (82–91 bp) and a partial sequence of exon 21 (81 bp). Similarly, the PCR amplification of the domain III fragment produced a partial sequence of exon 28 (81 bp), a full-length sequence of intron 28 (69–84 bp) and a partial sequence of exon 29 (165 bp) (Additional file 5: Fig. S2). In total, 23 and 41 haplotypes were identified from domain II and III, respectively (Additional file 6: Table S4). All haplotypes were centered on H2, suggesting that H2 is an ancestral sequence and that other haplotypes might have evolved from it (Additional file 7: Fig. S3; Additional file 8: Fig. S4).

**Fig. 4:**
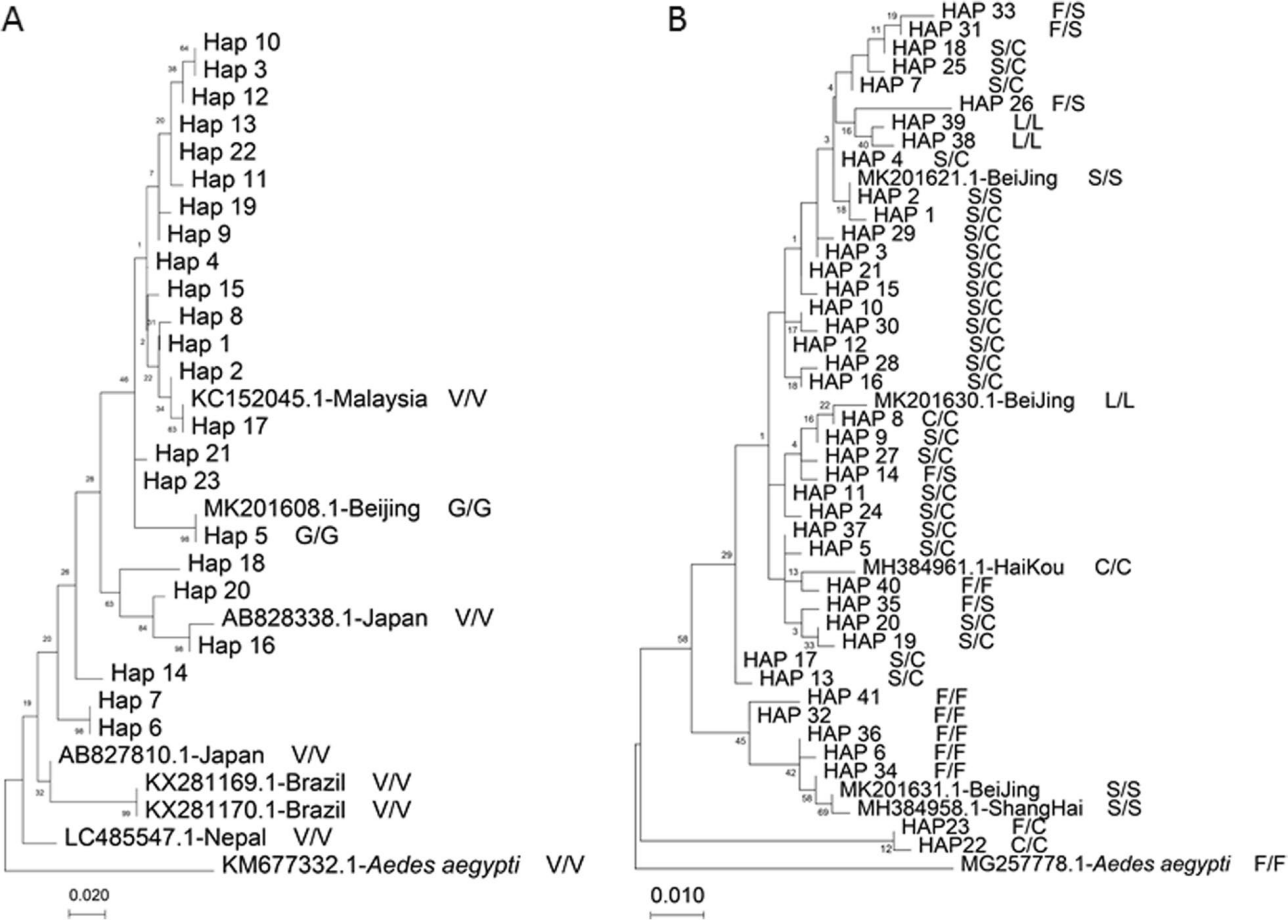
Phylogenetic tree of domains II and III of the VGSC gene in *Ae.* *albopictus* populations (Guangzhou). **a** Domain II, **b** domain III

Phylogenetic tree analysis of domain II showed that H5 of the G/G genotype was aggregated with the G/G genotype of *Ae.* *albopictus* from Beijing. H16 was aggregated with a strain of *Ae.* *albopictus* from Japan, whereas H17 aggregated with a strain of *Ae.* *albopictus* from Malaysia. The sequence of one *Ae.* *albopictus* strain from Nepal was independent from that of other *Ae.* *albopictus* strains, being the furthest related to other *Ae.* *albopictus* sequences. Two strains of *Ae.* *albopictus* from Brazil and another one from Japan clustered into a large branch (Fig. 4A).

Phylogenetic tree analysis of domain III showed that H22 and H23 clustered into one large branch, separated from other haplotypes. The haplotypes with the F/F genotype almost all clustered into a large branch, which also included sequences of *Ae.* *albopictus* from Beijing and Shanghai; however, their genotype was S/S. The last large branch was dominated by the S/C genotype; except for the genotype of H40, which was wild-type F/F, the genotypes of other haplotypes were mutant homozygous or heterozygous. Moreover, the large branch included a strain of *Ae.* *albopictus* from Haikou (C/C genotype) and two strains of *Ae.* *albopictus* from Beijing (L/L and S/S genotypes, respectively) (Fig. 4b).

## Discussion

From 2015 to 2019, a total of 3712 cases of dengue fever were reported in Guangzhou, including one death, with an average annual incidence of 5.14 cases per 100,000 population. The local cases were mainly concentrated in Liwan, Baiyun, Haizhu, Tianhe and Yuexiu districts (total of 2685 cases, 72.33%). Fewer cases were reported in surrounding areas, and thus Guangzhou could be divided into high-risk and low-risk dengue areas [44]. All of these five regions showed a high incidence of dengue fever, whereas the districts of Conghua, Panyu, Huadu, Huangpu, Nansha and Zengcheng did not show a high incidence of dengue fever [44].

Su et al. investigated fast emerging IR in *Ae.* *albopictus* collected from four districts in Guangzhou, reporting that *Ae.* *albopictus* adult populations in all four districts showed resistance to deltamethrin showed resistance [25]. In the present study, we found that the mutation frequency at locus 1016 was higher in mosquito populations collected in areas with a high incidence of dengue fever than in those with a low incidence (22.5% vs. 13.6%). We also found that the mutant gene frequency at locus 1016 was 12.3% and 7.1% in areas with and without a high incidence of dengue fever, respectively. Further, at the 1534 locus, the mutation frequency was 91.1% and 83.0% in areas with and without a high incidence of dengue fever, respectively, and the mutant gene frequency was 76.4% and 63.3%, respectively.

The F1534C and F1534S alleles were predominant in Tianhe District (high incidence area), and there were more individual mosquitoes with the mutant F1534C alleles (C/C + S/C) than with the wild-type mutant (F/C) alleles (46.9% vs 25.0%, respectively). Similarly, in this area, there were more individual mosquitoes with the F1534S mutant (S/S + S/C) alleles than with the wild-type/mutant (F/S) alleles (40.6% vs 9.4%, respectively). The trend shown by the F1534F, F1534S and F1534C alleles in Yuexiu District (high incidence area) was similar. The proportion of mutant individuals carrying genotype (S/S + C/C + S/C) was 57.2%, which was slightly more than that the proportion of wild-type homozygous and wild-type/mutant heterozygous individuals. In Zengcheng District (region without a high incidence of dengue fever), wild-type F1534F (45.8%) and mutant F1534S (47.9%) alleles were dominan; the prevalence of mutant F1534L and F1534C alleles was < 5%.

Li et al. evaluated multiple IR in urban *Ae.* *albopictus* populations in Guangzhou and found that the F1534S and F1534L mutations were significantly associated with deltamethrin resistance [26]. In that study, in the 15 wild-type populations of* Ae. albopictus* collected from 11 administrative regions of Guangzhou, F1534S was the dominant mutant genotype, which is consistent with the finding of Xu et al. [11]. On investigating the IR phenotype and* kdr* gene mutations in these populations, nonsynonymous mutations were found at locus 1016 of domain II and locus 1534 of domain III. V1016G was found at locus 1016 of domain II and F1534F, F1534S, F1534C and F1534L were found at locus 1534 of domain III. Although we identified multiple site mutations for the first time, further studies are warranted to confirm their relationship to IR. We did not conduct survival tests to investigate IR in this study, but other researchers in our department have performed such tests [25].

Previous studies on the malaria vector *Anopheles* mosquitoes have reported a strong association between* kdr* mutation frequency and phenotypic resistance status [26, 45, 46]. However, this association does not necessarily exist in all mosquito species. For example, *kdr* mutations were not identified in *Anopheles* *funestus* [26, 47] nor in some populations of *Anopheles* *sinensis *[48], even when WHO tube test mortality was very low (i.e. very high IR). The presence of* kdr* mutations, specifically the F1534 mutation, has been reported in *Ae.* *albopictus* in China and elsewhere [18, 23, 26, 27]. According to some studies, the F1534 mutation is associated with phenotypic resistance [11, 26, 49, 50], but conflicting results have been reported [26, 50].

Li et al. found that the F1534S mutation was associated with pyrethroid phenotypic resistance in *Ae.* *albopictus* in Hainan Province, China. However, the *kdr* mutation frequency was high in both resistant and susceptible individuals, indicating that the F1534S mutation is not completely predictive of resistance status [26, 49]. It is possible that regional differences exist in *kdr* mutations, as in the case of *An.* *sinensis* [26, 50]. Further, it has been widely reported that the F1534S, F1534C and F1534L mutations are associated with deltamethrin resistance [7, 11, 18, 26, 27, 49, 51, 52]. In past years in Guangzhou, China, the frequency of spraying insecticides in areas with a high prevalence of dengue was higher than that in those without a high prevalence of dengue, with the aim to control the prevalence of dengue and effectively manage mosquito vectors. Our results showed that the mutation frequency and mutant gene frequency in areas with a high incidence of dengue were higher than in thosein areas without a high incidence of dengue. Su et al*.* reported that *Ae.* *albopictus* populations have rapidly developed very high resistances to multiple commonly used insecticides at all study areas in Guangzhou [25]. Considering this variability, it is highly recommended that mosquito management strategies should be strengthened and that insecticide management should be standardized.

The haplotype frequencies were very much different in the CH and PY districts than in other districts, although PY was close to other sites. The frequency of the VC haplotype was significantly higher in high-risk dengue areas than in low-risk dengue areas. The phylogenetic tree analysis of domain II showed that H5 of the G/G genotype was aggregated with the G/G genotype of *Ae.* *albopictus* from Beijing. H16 was aggregated with a strain of *Ae.* *albopictus* from Japan and H17 with that from Malaysia. The sequence of one *Ae.* *albopictus* strain from Nepal was independent from that of others, being the furthest related to other *Ae.* *albopictus* sequences. Two strains of *Ae.* *albopictus* from Brazil and another one from Japan clustered into a large branch. Haplotype network analysis indicated that *kdr* mutations have multiple origins, showing at least three, four and six independent origins of* kdr* alleles in domain I, II and III of the VGSC gene, respectively. Multiple origins of* kdr* mutations is a common phenomenon in Culicinae mosquitoes and have been detected in *An.* *gambiae*, *An.* *sinensis* and *Ae.* *aegypti* [53–57]. In the present study, we found H2 to be the dominant haplotype, and almost all haplotypes were centered on H2, suggesting that H2 is an ancestral sequence and that other haplotypes might have evolved from it. Spatial heterogeneity of* kdr* mutations was found in the dengue vector *Ae.* *albopictus* in Guangzhou, China.

## Conclusions

We have delineated the distribution of* kdr* mutations in the dengue vector *Ae.* *albopictus* in Guangzhou, China. Amino acid substitutions at multiple sites (codons 1011, 1014, 1532, 1534, 1763, 1016) and three variations at codon 1534 were found. The mutation frequency and mutant gene frequency in areas with a high risk of dengue were higher than those in areas not at a high risk of dengue, suggesting that *Ae.* *albopictus* populations in some areas of Guangzhou have developed a certain degree of resistance. Our findings highlight the importance of monitoring and quantifying pyrethroid resistance level in field mosquitoes in urban districts. Limiting the spread of *kdr* alleles into rural areas should help prevent IR development.

## Supplementary Information

**Additional file 1: Table S1.** Information pertaining to *Ae.* *albopictus* sampling sites in Guangzhou.

**Additional file 2: Table S2.** PCR primers used to amplify DNA sequences of domains II, III, and IV of the VGSC gene.

**Additional file 3: Table S3.** Primers used for sequencing DNA sequences of domain II, III, and IV of the VGSC gene.

**Additional file 4: Figure S1.** Chromatogram showing nonsynonymous mutations in codons 1016 and 1534 of the VGSC gene in *Ae.* *Albopictus.* Note: Green peak represents adenine, red represents thymine, blue represents cytosine and black represents guanine.

**Additional file 5: Figure S2**. Schematic representation of the two regions of the *Ae.* *albopictus* VGSC gene analyzed in this study. The intron–exon structure between the predicted initiation and stop codons was identified based on genomic DNA and cDNA sequences.

**Additional file 6: Table S4.** Haploid gene accession number.

**Additional file 7: Figure S3.** Haplotype network diagram of domain II of the VGSC gene based on the medium joining network method.

**Additional file 8: Figure S4.** Haplotype network diagram of domain III of the VGSC gene based on the medium joining network method.

## Data Availability

The data sets supporting the results are included within the article and supporting information. Supporting information can be found in the online version of this article.

## Abbreviations

COI: Cytochrome* C* oxidase
kdr: Knockdown resistance
IR: Insecticide resistance
VGSC: Voltage-gated sodium channel
