# Supplementary material for: Spatial heterogeneity of knockdown resistance mutations in the dengue vector Aedesalbopictus in Guangzhou, China
Source: Parasit Vectors. 2022 May 3;15:156. doi: 10.1186/s13071-022-05241-7 (PMC9066732; doi:10.1186/s13071-022-05241-7)
Supplement: Supplementary file 1 — Additional file 1: Table S1. Information pertaining to Ae. albopictus sampling sites in Guangzhou. [file 13071_2022_5241_MOESM1_ESM.docx]

Table S1 Information about sampling sites of *Ae. albopictus* populations from Guangzhou

| Sampling sites | Dengue-risk* | Administrative  region |  | Environment | Coordinates  (°N, °E) | Date | Sample size | Area size | Resident population density |
| --- | --- | --- | --- | --- | --- | --- | --- | --- | --- |
| SMU | High  High | Bai Yun District |  | School | 23.194807, 113.343223 | 2020.11 |  |  |  |
| SNG |  |  |  | Residential District | 23.160589, 113.226654 | 2020.07 |  |  |  |
| TH | High | Tian He District |  | Residential District | 23.164606, 113.319323 | 2020.07 |  |  |  |
| NZHY | High | Hai Zhu District |  | Residential District | 23.076134, 113.310827 | 2020.07 |  |  |  |
| SS | High |  |  | Residential District | 23.11134, 113.284664 | 2020.08 |  |  |  |
| DJ | High | Li Wan District |  | Residential District | 23.085931, 113.232812 | 2020.08 |  |  |  |
| GY | High |  |  | Hospital | 23.123418, 113.240635 | 2020.10 |  |  |  |
| HX | High |  |  | Residential District | 23.086073, 113.250124 | 2020.10 |  |  |  |
| NS | low | Nan Sha District |  | Residential Park | 22.787836, 113.394568 | 2020.10 |  |  |  |
| YX | High | Yue Xiu District |  | Residential District | 23.140285, 113.266644 | 2020.10 |  |  |  |
| CH | Low | Cong Hua District |  | School | 23.561282, 113.600333 | 2020.10 |  |  |  |
| PY | Low | Pan Yu District |  | Rural Area | 22.954078, 113.345488 | 2020.10 |  |  |  |
| ZC | Low | Zeng Cheng District |  | Rural Area | 23.282149, 113.720136 | 2020.09 |  |  |  |
| HD | Low | Hua Du District |  | Rural Area | 23.346813, 113.237637 | 2020.11 |  |  |  |
| HP | Low | Huang Pu District |  | Rural Area | 23.312723, 113.586459 | 2020.11 |  |  |  |

Note: **SMU**: Southern Medical University, **SNG**: Songnan Pavilion , **TH**: Tianhe Villa, **NZHY**：Nanzhou Garden, **SS**：Su she, **DJ**: Dongyi sanqiaofang, **GY**： The Third Affiliated Hospital of Guangzhou Medical University, **NS**：Tanzhou Park, **YX**：Panfu community; **CH**: No. 7 middle school in Conghua district; **PY**: Xiaoping Village, **ZC**: Nangang Village, **HD**: Dongjing Village, **HP**: Xintian Village. * Cai et al 2020.

S1Table.

| Administrative Sample Proportion Administrative area Resident population density  Region size (N) (%) (square kilometers) (person / km2) |
| --- |
| Baiyun 69 9.77 795.79 3493  Haizhu 67 9.49 90.40 19073  Liwan 74 10.48 59.10 17124  Tianhe 42 5.95 96.33 18566  Yuexiu 59 8.36 33.80 35790  Conghua 109 15.44 1974.50 329  Panyu 86 12.18 529.94 3449  Huadu 43 6.09 970.04 1141  Huangpu 43 6.09 484.17 2378  Nansha 58 8.22 783.86 1016  Zengcheng 56 7.93 1616.47 780  Total 706 100 7434.40 2059 |
