# Supplementary material for: Spatial heterogeneity of knockdown resistance mutations in the dengue vector Aedesalbopictus in Guangzhou, China
Source: Parasit Vectors. 2022 May 3;15:156. doi: 10.1186/s13071-022-05241-7 (PMC9066732; doi:10.1186/s13071-022-05241-7)
Supplement: Supplementary file 2 — Additional file 2: Table S2. PCR primers used to amplify DNA sequences of domains II, III, and IV of the VGSC gene. [file 13071_2022_5241_MOESM2_ESM.docx]

Table S2. List of primers for PCR amplification of DNA sequences of domain Ⅱ, Ⅲ, Ⅳ in VGSC gene in this study

| Fragments Direction The name Sequence（5'- 3'） Length  of the primer |
| --- |
| Forward aegSCF20 GACAATGTGGATCGCTTCCC 480bp  Domain Ⅱ Reverse aegSCR21 GCAATCTGGCTTGTTAACTTG  Forward aegSCF7 GAGAACTCGCCGATGAACTT 720bp  Domain Ⅲ Reverse aegSCR7 GACGACGAAATCGAACAGGT  Forward albSCF6 TCGAGAAGTACTTCGTGTCG 280bp  Domain IV Reverse albSCF8 AACAGCAGGATCATGCTCG |
