# Supplementary material for: Spatial heterogeneity of knockdown resistance mutations in the dengue vector Aedesalbopictus in Guangzhou, China
Source: Parasit Vectors. 2022 May 3;15:156. doi: 10.1186/s13071-022-05241-7 (PMC9066732; doi:10.1186/s13071-022-05241-7)
Supplement: Supplementary file 3 — Additional file 3: Table S3. Primers used for sequencing DNA sequences of domain II, III, and IV of the VGSC gene. [file 13071_2022_5241_MOESM3_ESM.docx]

Table S3. List of primers for Sequencing of DNA sequences of domain Ⅱ, Ⅲ, Ⅳ in VGSC gene in this study

| Fragments Direction The name Sequence（5'- 3'） Length  of the primer |
| --- |
| Forward aegSCF3 GTGGAACTTCACCGACTTCA  Domain Ⅱ Reverse aegSCR22 TTCACGAACTTGAGCGCGTTG 380bp  Domain Ⅲ Reverse aegSCR8 TAGCTTTCAGCGGCTTCTTC 380bp  Domain IV Forward albSCF7 AGGTATCCGAACGTTGCTGT 280bp |
