# Supplementary figures and images for: Spatial heterogeneity of knockdown resistance mutations in the dengue vector Aedesalbopictus in Guangzhou, China
Source: Parasit Vectors. 2022 May 3;15:156. doi: 10.1186/s13071-022-05241-7 (PMC9066732; doi:10.1186/s13071-022-05241-7)

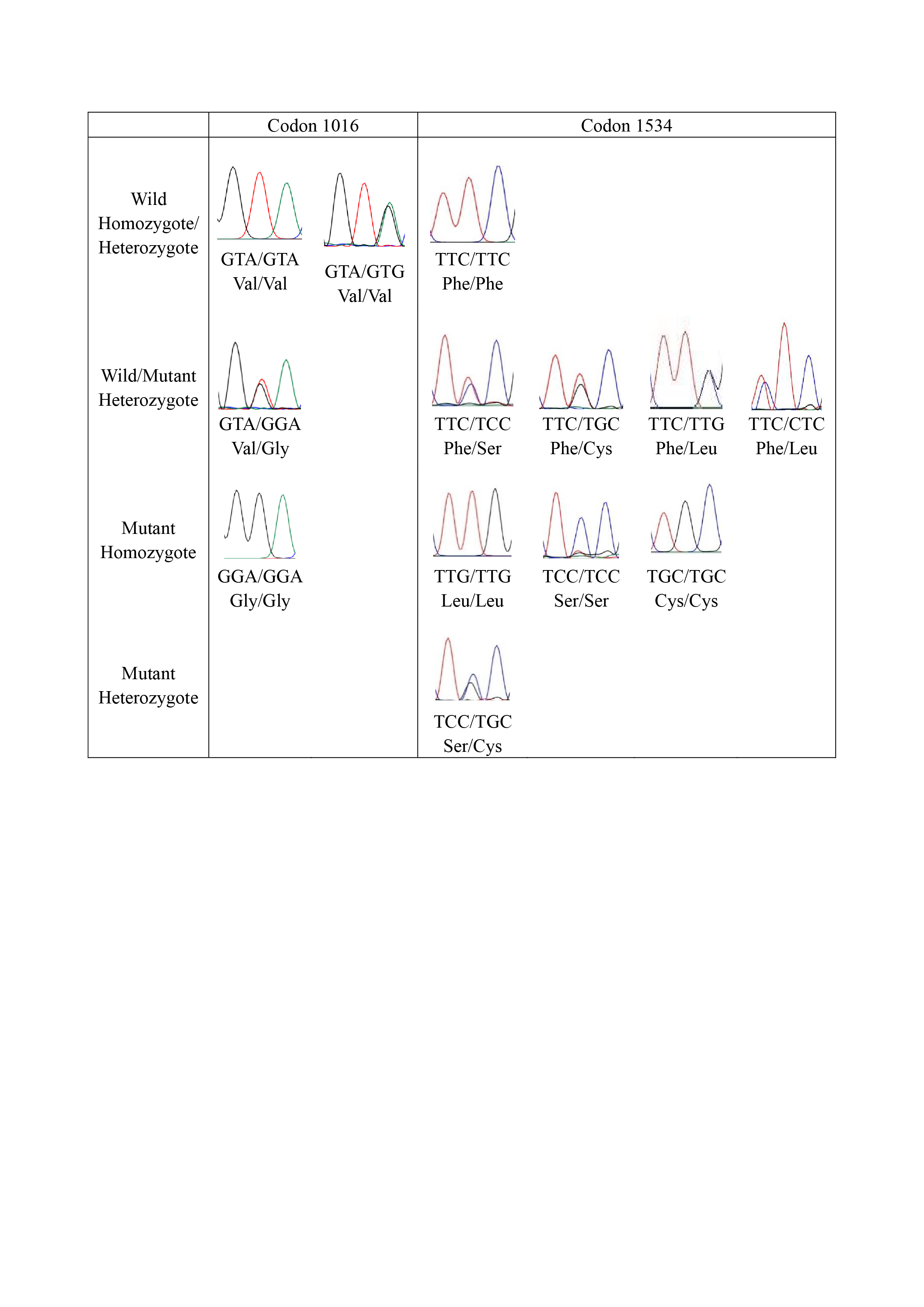

Supplement: Supplementary file 4 — Additional file 4: Figure S1. Chromatogram showing nonsynonymous mutations in codons 1016 and 1534 of the VGSC gene in Ae. Albopictus. Note: Green peak represents adenine, red represents thymine, blue represents cytosine and black represents guanine. [file 13071_2022_5241_MOESM4_ESM.tif]

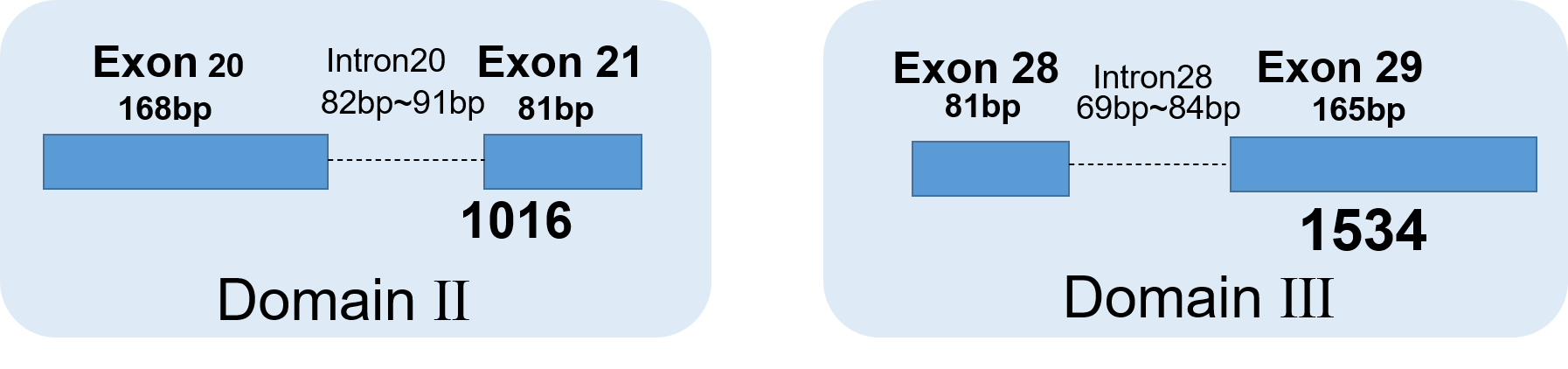

Supplement: Supplementary file 5 — Additional file 5: Figure S2. Schematic representation of the two regions of the Ae. albopictus VGSC gene analyzed in this study. The intron–exon structure between the predicted initiation and stop codons was identified based on genomic DNA and cDNA sequences. [file 13071_2022_5241_MOESM5_ESM.png]

## Slide 1
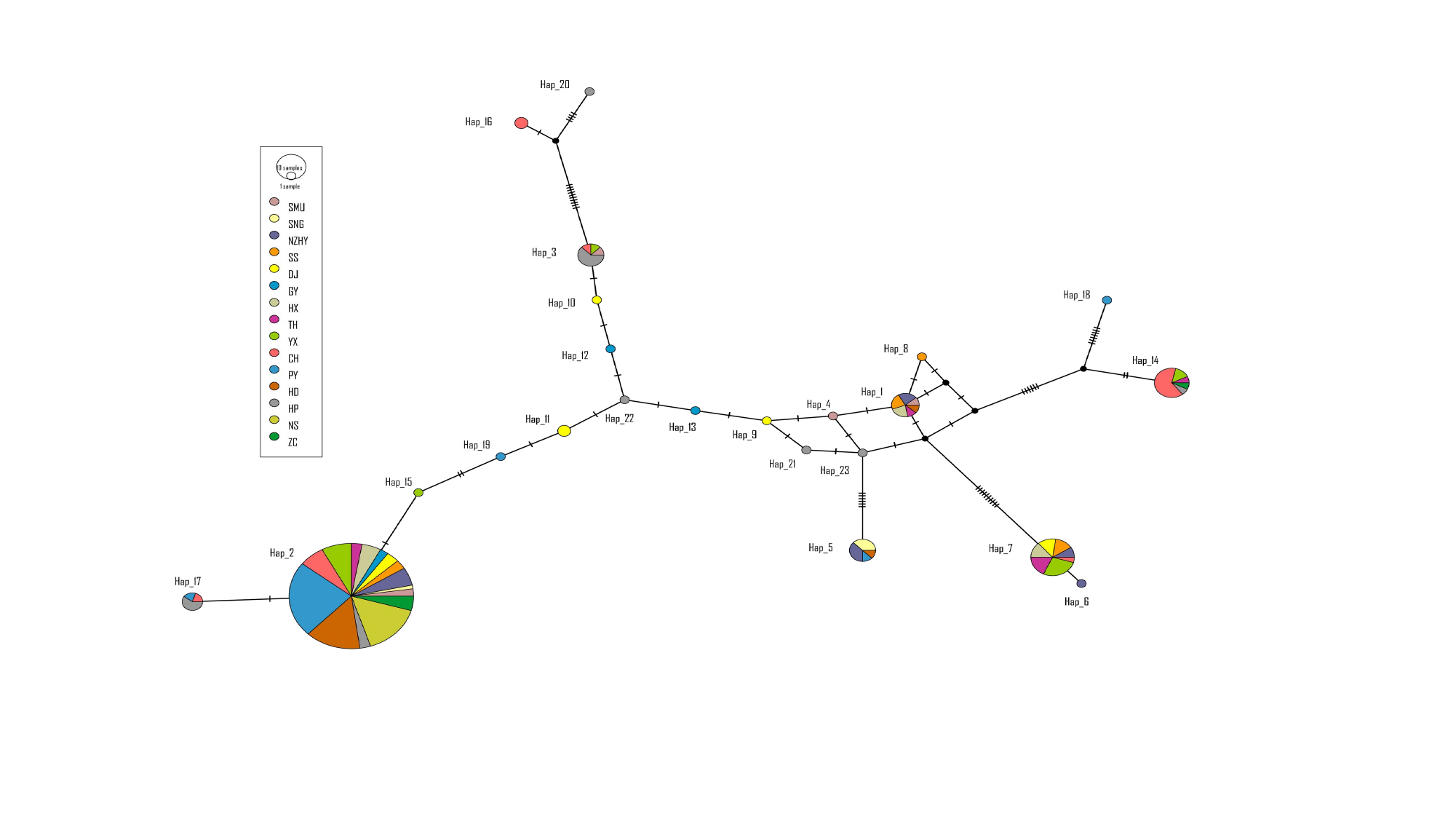

Supplement: Supplementary file 7 — Additional file 7: Figure S3. Haplotype network diagram of domain II of the VGSC gene based on the medium joining network method. [file 13071_2022_5241_MOESM7_ESM.pptx]

## Slide 1
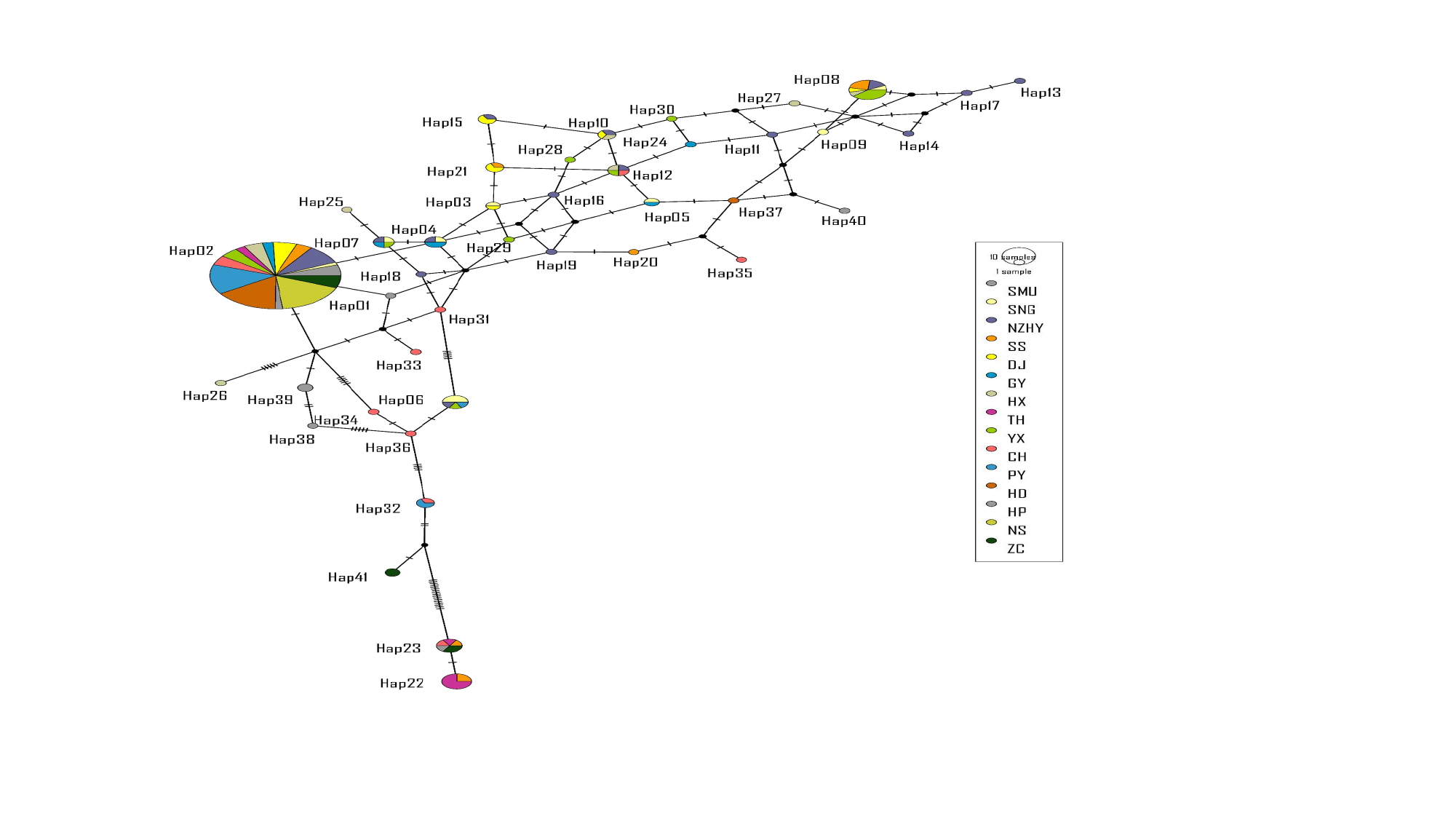

Supplement: Supplementary file 8 — Additional file 8: Figure S4. Haplotype network diagram of domain III of the VGSC gene based on the medium joining network method. [file 13071_2022_5241_MOESM8_ESM.pptx]
