# Supplementary material for: Spatial heterogeneity of knockdown resistance mutations in the dengue vector Aedesalbopictus in Guangzhou, China
Source: Parasit Vectors. 2022 May 3;15:156. doi: 10.1186/s13071-022-05241-7 (PMC9066732; doi:10.1186/s13071-022-05241-7)
Supplement: Supplementary file 6 — Additional file 6: Table S4. Haploid gene accession number. [file 13071_2022_5241_MOESM6_ESM.docx]

Table S4

| domain II | | | |  |
| --- | --- | --- | --- | --- |
| haplotype | Genbank | haplotype | Genbank |  |
| d2h1 | MZ823334 | d2h13 | MZ823322 |  |
| d2h2 | MZ823333 | d2h14 | MZ823321 |  |
| d2h3 | MZ823332 | d2h15 | MZ823320 |  |
| d2h4 | MZ823331 | d2h16 | MZ823319 |  |
| d2h5 | MZ823330 | d2h17 | MZ823318 |  |
| d2h6 | MZ823329 | d2h18 | MZ823317 |  |
| d2h7 | MZ823328 | d2h19 | MZ823316 |  |
| d2h8 | MZ823327 | d2h20 | MZ823338 |  |
| d2h9 | MZ823326 | d2h21 | MZ823337 |  |
| d2h10 | MZ823325 | d2h22 | MZ823336 |  |
| d2h11 | MZ823324 | d2h23 | MZ823335 |  |
| d2h12 | MZ823323 |  |  |  |
| domain III | | | |  |
| haplotype | Genbank | haplotype | Genbank |  |
| d3h1 | MZ823284 | d3h23 | MZ823303 |  |
| d3h2 | MZ823283 | d3h24 | MZ823302 |  |
| d3h3 | MZ823282 | d3h25 | MZ823301 |  |
| d3h4 | MZ823281 | d3h26 | MZ823300 |  |
| d3h5 | MZ823280 | d3h27 | MZ823299 |  |
| d3h6 | MZ823279 | d3h28 | MZ823298 |  |
| d3h7 | MZ823278 | d3h29 | MZ823297 |  |
| d3h8 | MZ823277 | d3h30 | MZ823296 |  |
| d3h9 | MZ823276 | d3h31 | MZ823295 |  |
| d3h10 | MZ823275 | d3h32 | MZ823294 |  |
| d3h11 | MZ823315 | d3h33 | MZ823293 |  |
| d3h12 | MZ823314 | d3h34 | MZ823292 |  |
| d3h13 | MZ823313 | d3h35 | MZ823291 |  |
| d3h14 | MZ823312 | d3h36 | MZ823290 |  |
| d3h15 | MZ823311 | d3h37 | MZ823289 |  |
| d3h16 | MZ823310 | d3h38 | MZ823288 |  |
| d3h17 | MZ823309 | d3h39 | MZ823287 |  |
| d3h18 | MZ823308 | d3h40 | MZ823286 |  |
| d3h19 | MZ823307 | d3h41 | MZ823285 |  |
| d3h20 | MZ823306 |  |  |  |
| d3h21 | MZ823305 |  |  |  |
| d3h22 | MZ823304 |  |  |  |
